# The Rhythmic Embodied Perception Framework of breath, brain, and perception

**DOI:** 10.1093/nc/niag030

**Published:** 2026-06-22

**Authors:** Ralph Andrews, Paul M Dockree

**Affiliations:** Trinity Institute of Neuroscience, Trinity College Dublin, Lloyd Building, 42A Pearse St, Dublin D02 R123, Ireland; Trinity Institute of Neuroscience, Trinity College Dublin, Lloyd Building, 42A Pearse St, Dublin D02 R123, Ireland

**Keywords:** awareness, embodiment, metacognition, perception, philosophy, theories and models

## Abstract

Breathing, beyond its metabolic role, has been increasingly implicated in shaping perception, attention, and aspects of cognitive processing. Here, we propose the Rhythmic Embodied Perception (REP) Framework, a multi-level framework linking body and brain to explain how respiration structures conscious experience. At the neurophysiological level, slow respiratory cycles modulate cortical oscillations *via* cross-frequency coupling, biasing excitability patterns across frontoparietal and occipital networks. At the perceptual–phenomenological level, these oscillatory dynamics create transient pause states—low-excitability phases of alpha/theta activity—during which sensory input is most likely to reach awareness. Respiration rhythmically gates these windows, coordinating perceptual clarity and attentional receptivity. At the regulatory-attentional level, volitional breath control stabilizes oscillatory and perceptual dynamics by entraining neuromodulatory networks such as the locus coeruleus-noradrenaline system, extending pause duration, and enhancing attentional stability. The REP Framework yields concrete, testable predictions: for example, respiratory phase should modulate phase-dependent sensory detection, and slow-paced breathing should strengthen respiration–cortex coupling and improve attentional continuity. Conceptually, this framework unifies evidence from neuroscience, theories of discrete perception, and yogic models of prana, grounding ancient accounts of pulsative attention in contemporary neural dynamics. Practically, it positions respiration as a trainable lever for shaping perceptual and attentional rhythms, offering an experimentally tractable route to probe the embodied mechanisms of consciousness. By highlighting the rhythmic interplay of breath, brain, and perception, the REP Framework provides a novel, integrative perspective on how bodily rhythms structure conscious experience, with implications for cognitive neuroscience, contemplative practice, and therapeutic interventions.

Highlights

- Proposes the Rhythmic Embodied Perception (REP) Framework, linking respiration, neural oscillations, and perceptual dynamics within a unified model of consciousness.
- Identifies respiration as a global rhythmic scaffold that modulates cortical excitability, attention, and perceptual sampling.
- Integrates neurophysiological evidence with phenomenological and contemplative perspectives, providing a cross-disciplinary account of embodied consciousness.
- Generates testable predictions connecting breathing patterns, locus coeruleus activity, oscillatory brain dynamics, and perceptual stability.
- Positions breath regulation as a mechanistic bridge between first-person experience and neural processes, offering new directions for experimental consciousness research.

## Introduction

The rhythm of breath is more than a metabolic necessity—it is a dynamic force that shapes our moment-to-moment experience of the world. Recent studies in cognitive neuroscience have demonstrated that perception and cognitive performance fluctuate with the phase of the respiratory cycle. Across sensory modalities, task performance has been shown to vary depending on whether a stimulus coincides with inhalation or exhalation. These effects span from enhanced detection of near-threshold stimuli in the visual and tactile domains (Kluger et al. 2021; Grund et al. 2022) to fluctuations in memory (Zelano et al. 2016; Nakamura et al. 2018, 2022), pain sensitivity (Arsenault et al. 2013; Iwabe et al. 2014), and emotional processing (Zelano et al. 2016). In addition, behavioural timing itself becomes entrained to respiration, with task-relevant events preferentially aligned to specific respiratory phases (Huijbers et al. 2014; Melnychuk et al. 2018; Perl et al. 2019; Grund et al. 2022; Johannknecht and Kayser 2022). At the neural level, respiratory phase, rate, and depth modulate cortical oscillations across multiple frequency bands (Herrero et al. 2018; Kluger and Gross 2020, 2021). Collectively, these findings suggest that respiration acts as a mediator between external demands and internal psycho-physiological states, coordinating bodily rhythms with perceptual, cognitive, and motor processes.

Perception also fluctuates at much faster timescales, in line with ongoing cortical oscillations in the theta and alpha ranges (VanRullen 2016, 2018; Fiebelkorn and Kastner 2019; Raposo et al. 2023). These rhythms are associated with the frontoparietal network (Marek and Dosenbach 2018) and are thought to underpin the discrete nature of attentional sampling, whereby perceptual access emerges rhythmically rather than continuously. Thus, we observe two rhythmic scaffolds of experience: a slower, body-driven rhythm of respiration, and a faster, cortex-driven rhythm of attention. While these have typically been studied separately, they may in fact represent interacting layers of an embodied system for regulating consciousness.

Research in psychology and cognitive neuroscience has increasingly recognized the value of contemplative traditions for theoretical models of consciousness and attention. For example, Sedlmeier and Srinivas (2016) review how classical yoga practices can influence cognitive processes and emotional regulation, offering a framework for studying mental training effects on attention, awareness, and perceptual stability. Tripathi and Bharadwaj (2021) draw on yogic concepts to elucidate meditation-related changes in perceptual and neural dynamics, highlighting how ancient frameworks can inform contemporary theories of brain–mind relations. More recently, Katyal (2022) integrates first-person yogic phenomenology with neuroscientific models of rhythmic attention, arguing that embodied practices like meditation and breath regulation reveal structured temporalities of conscious experience. These works establish a scholarly precedent for integrating yogic philosophy with empirical and theoretical research on consciousness, grounding the present REP Framework within a broader, academically rigorous discourse on how contemplative insights can contribute to scientific models of perception and attention.

Within yogic psychology, breath is understood to regulate pranah, the vital force said to govern attention, perception, and mental stability (Práńáyáma, Anandamurti 1999; Práńáyáma Sádhaná, Anandamurti 1958). Pranic flow is described as inherently pulsative, implying that perceptual experience itself unfolds in discrete rhythmic intervals. Practices of pranayama (the regulation of prana) aim to stabilize these pulsations, thereby refining attention and facilitating meditative states. Strikingly, these phenomenological claims parallel contemporary neuroscientific observations of respiratory modulation of neural oscillations and perception, yet the two traditions have rarely been brought into systematic dialogue.

Here, we propose the Rhythmic Embodied Perception (REP) Framework, that integrates multi-level models and unifies their converging lines of evidence. The REP Framework conceptualizes respiration as a rhythmic scaffold linking body and brain across three tightly coupled levels. First, at the neurophysiological level, respiration modulates large-scale neural oscillations, biasing cortical excitability over time. Second, at the perceptual–phenomenological level, these oscillatory modulations manifest as discrete fluctuations in perceptual clarity, including transient pause states described in contemplative traditions. Third, at the regulatory–attentional level, voluntary control of breathing stabilizes these oscillatory–perceptual dynamics *via* neuromodulatory systems such as the locus coeruleus (LC). Each section of this review addresses one level of the REP Framework, and the Discussion integrates them into a unified account of embodied conscious perception.

From a yogic perspective, prana is often described as a fundamental organizing principle of the material and cognitive world. While this ontological claim is profound, it is not the central argument of this article. Instead, we treat prana as a heuristic analogue, drawing on millennia of first-person insight to illuminate how subjective experience may align with neural excitability and respiratory rhythms. By incorporating pranic concepts in this way, we broaden the discussion, highlighting phenomenological perspectives that resonate with contemporary neuroscientific observations, without making metaphysical claims.

By synthesizing neuroscientific research on respiratory modulation of perception, theories of discrete perceptual sampling, and yogic models of pranic regulation, this review positions breath as a rhythmic interface between body and brain—one that shapes perceptual access and conscious experience itself. This framework generates concrete, testable predictions, and offers a principled approach for studying how embodied rhythms and volitional breath control may tune the neurophysiological mechanisms underlying fluctuations in perceptual clarity and conscious access.

## Breath as a modulator of perception, attention, and neural oscillations

Over the past decade, cognitive neuroscience has provided converging evidence that respiratory phase modulates perceptual and cognitive performance. Across a wide range of sensory–cognitive paradigms, task outcomes depend on whether critical events coincide with inhalation or exhalation. Early demonstrations by Zelano et al. (2016) showed that memory retrieval and emotional face discrimination were enhanced during inhalation. Subsequent studies have replicated respiratory phase effects across visual perception (Flexman et al. 1974; Kluger et al. 2021), visuospatial processing (Perl et al. 2019), oddball detection (Melnychuk et al. 2018), memory retrieval (Nakamura et al. 2018, 2022), perceptual awareness (Molle et al. 2023), numerical estimation (Belli et al. 2021), pain perception (Arsenault et al. 2013), voluntary motor timing (Park et al. 2020), and reaction times across multiple sensory domains (Johannknecht and Kayser 2022).

Task performance in these studies relies on a variety of sensory, cognitive, and motor processes. While these studies have repeatedly supported the notion that the respiratory cycle influences task performance in a wide-ranging manner, it is not precisely clear which processes are being modulated by respiratory phase. In line with the focus of the present review, there are two noteworthy studies which have attempted to isolate the effect of respiratory phase on sensory perceptual sensitivity: a study from Kluger et al. (2021) on visual perception and alpha brain activity, and Grund et al. (2022) on tactile perception and further links to the cardiac cycle.

**Figure 1:**
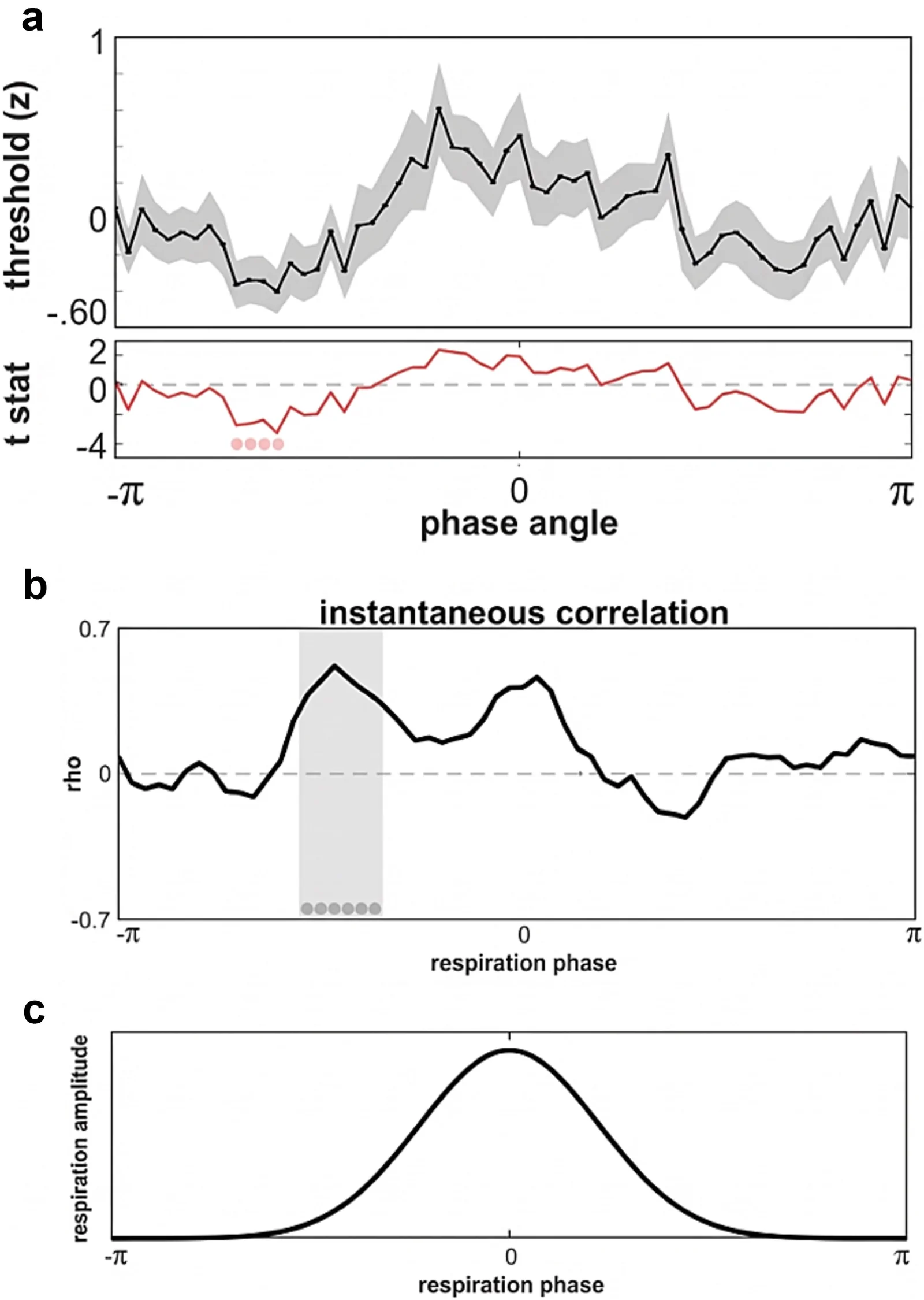
Adapted from Kluger et al. (2021). Visual detection thresholds fluctuate with the respiration cycle and correlate with alpha power. (a) Group-level normalized psychometric function (PsychF) thresholds across respiration phase angles (mean ± SEM, top) and corresponding *t*-values (bottom), showing a significant decrease during inspiration (−128° to −76°, red dots). (b) Instantaneous group-level correlation between alpha power (8–13 Hz) and PsychF thresholds, with significant clusters marked in grey. (c) Respiration phase reference plot.

Kluger et al. (2021) applied a task which asked participants to detect visual stimuli that were near the threshold of perceptibility. The authors found that phases around mid-inhalation were when the perceptual threshold was lowest (most sensitive). Analysis of oscillatory brain activity using magnetencephalography (MEG), showed that amplitude in the alpha range (8–13 Hz) in parieto-occipital areas were reduced over these same mid-inhalation phases. There were correlations between the perceptual threshold and alpha power over mid-inhalation phases (Fig. 1). The authors interpret their findings as a modulation in neuronal excitability in the visual system *via* changes in alpha over these inhalation phases. They indicate that this effect could be attributed to an ‘active sensing’ process of actively drawing in external information which is organized by respiratory dynamics. Similarly, others have suggested that the dependency of olfaction on the respiratory dynamics may orchestrate sensory reception more globally (Perl et al. 2019).

Complementing this work, Kluger and Gross (2021) demonstrated that respiration modulates neural oscillatory amplitude at rest across a broad frequency range and distributed cortical regions, a phenomenon termed respiratory-modulated brain oscillations. Respiratory phase–amplitude coupling varied systematically across brain areas and overlapped with canonical resting-state networks, including default mode, dorsal attention, and salience networks. This indicates that respiration shapes large-scale neural dynamics even in the absence of explicit task demands.

Respiratory phase modulation of behaviour is concurrent with a modulation of supporting brain oscillations (Zelano et al. 2016; Perl et al. 2019; Kluger et al. 2021; Nakamura et al. 2022). Alpha oscillation power in visual-related areas show a negative correlation with visual target detectability (Davidson et al. 2022). They are thought to represent a function of sensory inhibition which aids in distractor suppression but potentially inhibits target detection (Gutteling et al. 2022; Jensen 2024). Thus, the finding of Kluger et al. about a respiratory phase modulation of alpha amplitude is interpreted by the authors as an adaptive attuning of neural networks to optimally facilitate the signal-to-noise ratio of subtle visual information. Specifically, alpha power is thought to represent the degree of cortical excitability (magnitude of response to stimulation) in visual processing areas (Dinse et al. 2023). This conclusion is in alignment with another analysis from Kluger and colleagues which demonstrated a respiratory-cyclic modulation of global aperiodic neural activity, a proxy measure of excitation-to-inhibition ratio (Kluger et al. 2023b; Kluger et al. 2025).

Grund et al. (2022) assessed perceptual sensitivity in the tactile domain by applying near-threshold electrical stimulation to the finger. Detectability was analysed as a function of both the respiratory cycle and the cardiac cycle (R–R interval), which are intrinsically coupled *via* respiratory sinus arrhythmia (Yasuma and Hayano 2004; Grossman 2024). During inhalation, pulmonary stretch receptors engage brainstem pathways that reduce vagal efferent input to the heart, increasing heart rate, whereas during exhalation vagal activity increases and heart rate decreases. This coupling is thought to optimize gas exchange during inhalation while permitting cardiac relaxation during exhalation.

**Figure 2:**
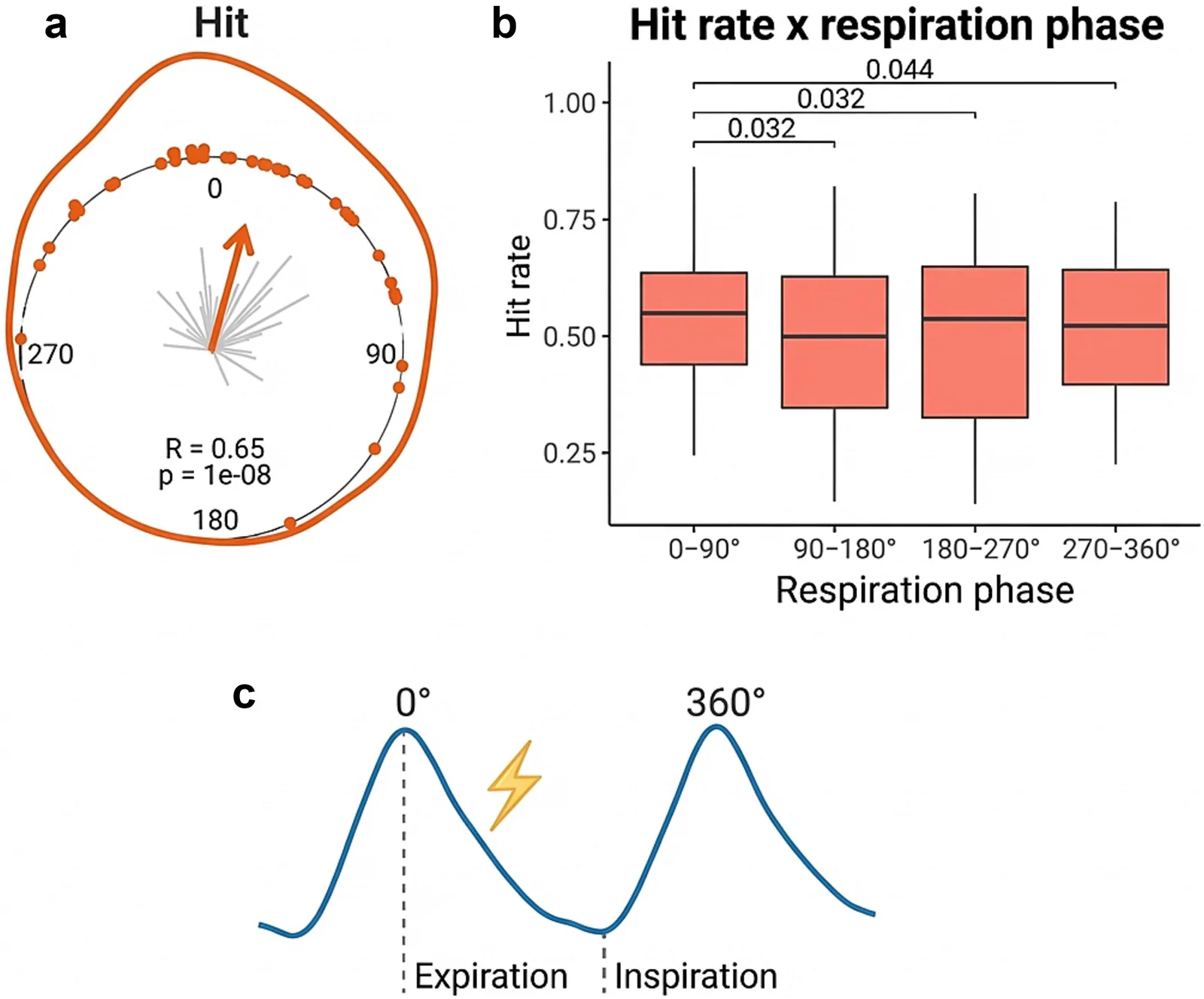
Adapted from Grund et al. (2022). Tactile stimuli occurred mostly around exhalation onset, with higher detection in the first quadrant of the respiratory cycle. (a) Circular distribution of mean stimulus onsets for ‘hit’ trials; zero degrees marks expiration onset. Dots show each participant’s mean angle, grey lines indicate intra-individual variability, and the central arrow shows the group mean (length = overall mean resultant length, R). The outer line shows the density distribution; Rayleigh test p-value is below. (b) Detection rates across four respiratory quadrants; lines above boxplots indicate significant pairwise t-tests after FDR correction. (c) Respiration phase in degrees for reference.

Participants preferentially aligned their respiratory cycle such that tactile stimulation occurred from late inhalation to early exhalation. Moreover, the degree of respiratory phase-locking positively correlated with detection rates, with highest sensitivity occurring during early exhalation (Fig. 2). With respect to the cardiac cycle, correct detection trials were more likely to occur during diastole, the phase of cardiac relaxation. In addition, both correct detections and correct rejections were followed by cardiac deceleration, expressed as prolonged diastolic intervals in subsequent heartbeats.

These findings suggest that respiratory and cardiac rhythms are adaptively coordinated to optimize tactile perception. Specifically, late inhalation to early exhalation may coincide with elevated arousal and attentional vigilance, while the moment of perceptual detection benefits from diastole, when cardiac interoceptive signalling is reduced and thus less likely to interfere with external tactile information. This pattern points to a trade-off between arousal optimization and transient interoceptive quiescence in supporting subtle sensory detection. Notably, post-trial confidence ratings revealed greater diastolic prolongation following unconfident compared with confident trials, potentially reflecting uncertainty-driven autonomic adjustment to enhance subsequent vigilance.

Perception is necessarily supported by attention, which itself fluctuates over time and is associated with activity within frontoparietal control networks and their interaction with default mode regions (Newton et al. 2010; Spreng et al. 2010; Gao and Lin 2011; Xin and Lei 2015; Marek and Dosenbach 2018; Koshino et al. 2023; Liu et al. 2023).

A dynamical systems account proposed by Melnychuk et al. (2018, 2021) situates respiration within this attentional architecture *via* the LC, a brainstem nucleus central to noradrenergic modulation of arousal and attentional gain (Aston-Jones and Cohen 2005). The LC both receives respiratory-related inputs—from the preBötzinger complex (Yackle et al. 2017), CO_2_ chemoreceptors (Pineda and Aghajanian 1997; Biancardi et al. 2008; Quintero et al. 2017), and possibly *via* vagal afferents through the nucleus solitary tract (Mello-Carpes and Izquierdo 2013; Hulsey et al. 2017; Gerritsen and Band 2018)—and modulates cortical excitability through widespread projections (Berridge and Waterhouse 2003; Poe et al. 2020; Waterhouse et al. 2022). Coupling between respiratory rhythms, LC activity, and cortical oscillations may therefore bias attentional stability or reorientation over time. These interconnected sources of oscillations, the LC, cortical attentional networks, and respiration, are considered to be inherently coupled and capable of entraining synchronization in each other (Melnychuk et al. 2018, 2021). Switching between periods of inter-oscillatory harmony and disharmony could underpin attentional fluctuations, primed towards a task or task-unrelated mind wandering.

This theoretical model has received positive initial empirical support. Melnychuk et al. (2018) demonstrated that indeed, activity in the LC shows synchronization with the respiratory cycle in humans. This was observed for both fMRI BOLD activity from the LC as well as utilizing pupil diameter as a proxy measurement (Murphy et al. 2014; Joshi et al. 2016; DiNuzzo et al. 2019; Bang et al. 2023; Meissner et al. 2023). Furthermore, Melnychuk et al. (2021) concluded that there was significant information transfer (Granger causality) between pupil diameter, respiration, and theta:beta ratio in a frontal cortical area, with the latter used to represent cortically-supported attentional engagement (Angelidis et al. 2016). Outside of this model, further validation comes from demonstrations that respiratory dynamics and pupillary dynamics are linked (Schaefer et al. 2023, 2025; Kluger et al. 2024), thus enforcing the idea of an inherent coupling to the LC.

The present authors addressed a prediction of this model: does slowing down and pacing the respiratory oscillation push the dynamical system into stability, reducing fluctuations in LC activity and attentional state? This study found that slowing down respiration to 0.1–0.15 Hz resulted in dominant pupillary oscillations at the same rate, and further, a reduction in behavioural response errors (Andrews et al. 2025). Thus, these findings demonstrate the potential for respiratory dynamics to alter LC dynamics and the stability of attentional state. A more detailed account of the study is provided in the final *Discussion* section, alongside other studies addressing the effects of slow-paced breathing.

Together, these findings indicate that respiration modulates perception and attention across multiple timescales by shaping cortical excitability, autonomic state, and neuromodulatory gain. Sensory detection is enhanced during specific respiratory phases through coordinated adjustments in neural oscillations and interoceptive signalling, while attentional stability appears sensitive to the strength of coupling between respiratory rhythms and LC-mediated gain control. These observations establish respiration as a central organizing rhythm linking bodily physiology to perceptual and attentional dynamics.

## Perception is discretized by neural oscillations

The temporal nature of perception has seemingly always been an actively debated topic: do we perceive continuously or in discrete moments? Philosophers from Zeno (Arrow paradox, *Zeno’s Paradoxes*), to Aristotle (Bowin 2017), to William James (James 1890) have pondered this question. Recent advances in the neuroscience of perception have yielded some highly intriguing and consistent findings which support the view that perceptual access fluctuates rhythmically rather than continuously.

This field of research has been spearheaded by Professor VanRullen who has written comprehensive reviews on discrete perception which are highly recommended for an in-depth discussion of studies that fall under this topic (VanRullen 2016, 2018; see also Fiebelkorn and Kastner 2019; Keitel et al. 2022). From these studies, there are two main areas of supporting evidence.

**Figure 3:**
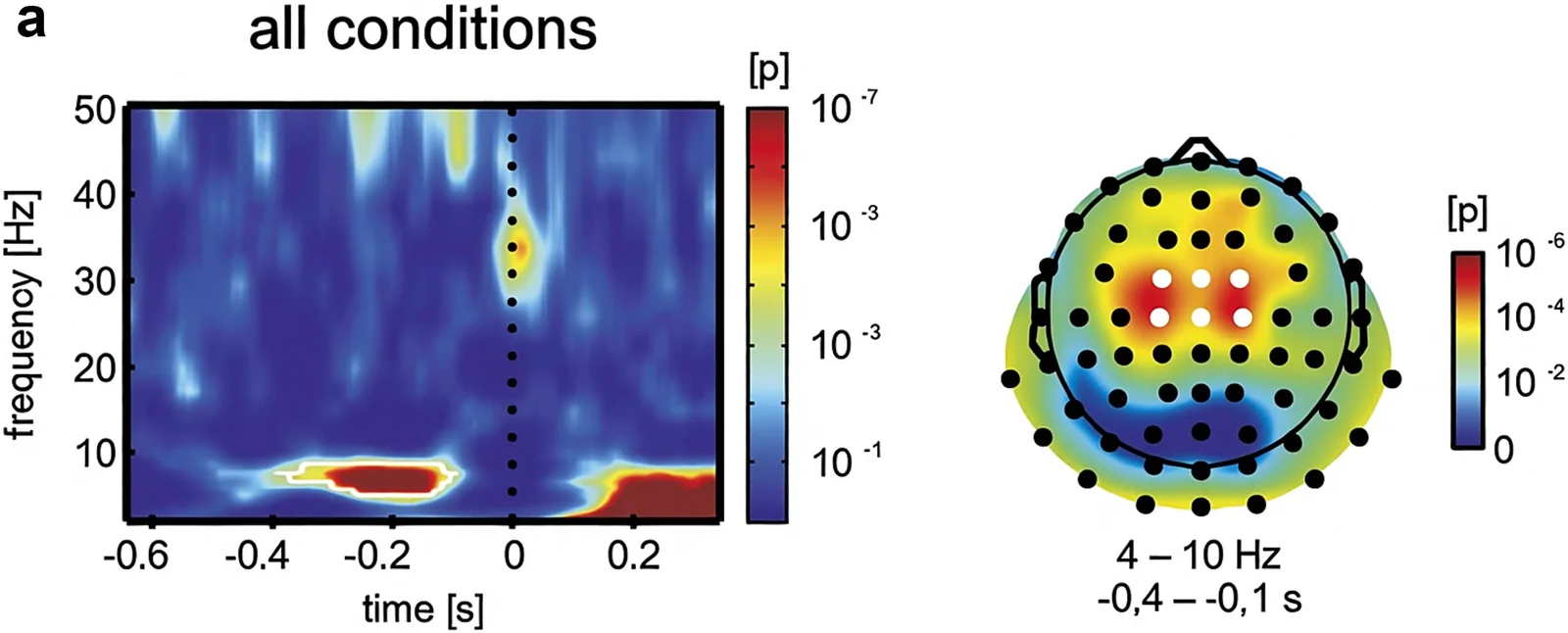
Adapted from Busch and VanRullen (2010). Pre-stimulus EEG phase predicts the strength of stimulus-evoked responses. Circular–linear correlations between EEG phase and post-stimulus global field power were computed across all channels and conditions. The colour scale shows p-values, with significant clusters outlined in white (FDR 5%). Strongest correlations occurred near 7 Hz during the −400 to −100 ms pre-stimulus window and at frequencies <5 Hz shortly after stimulus onset. The scalp map shows correlation distribution for 4–10 Hz within the −400 to −100 ms interval, with white dots marking ROI channels.

First, an individual’s perception of a stimulus depends on the phase of ongoing brain oscillations at the time of the stimulus presentation, as measured by electroencephalography (EEG). For example, Busch and VanRullen (2010) had participants detect near-threshold visual stimuli whilst monitoring EEG. They observed a robust response in the EEG when participants declared detection of the stimulus. The degree of this response as well as the declaration of detection was highly correlated to the phase of 7 Hz brain activity which was occurring approximately 200 ms before the stimulus appeared (Fig. 3). In other words, successful perception and the corresponding neural response depended on the phase of the brain wave at the time of the stimulus. A number of studies have echoed this finding, that perception of a visual stimulus depends on the phase of the brain oscillation immediately preceding the presentation of the stimulus. The large majority of studies have reported this perceptual sampling rhythm to be <15 Hz, with high convergence on 7 and 11 Hz (see VanRullen 2016, 2018 for comprehensive list). VanRullen suggests here that these two distinctly reoccurring frequencies could be related to frontocentral-mediated visual attention at 7 Hz, and occipital-mediated sensory visual perception at 11 Hz.

An additional avenue of supporting research for discrete perception has shown that the behavioural response to a stimulus also exhibits fluctuations on this timescale. In these experiments, a pre-stimulus cue is given which is intended to ‘reset’ attention, and then the target stimulus is presented after variable intervals. The proceeding behavioural response, either a reaction time (speed) or declaring detection (detectability) is relatively better or worse depending on the cue-to-stimulus time interval. As an example for clarity, responses given after a 300 ms cue-to-stimulus interval could show consistent fast reaction times, whereas those given after a 450 ms interval could show consistent slow reaction times. Plotting these behavioural time courses over several hundred milliseconds produces a waveform with a periodicity of around 7 Hz (VanRullen 2016, 2018).

It has further been seen that the frequency in the behavioural time course reduces to 3–4 Hz when there are targets being cued at two locations (Fiebelkorn et al. 2013; Huang et al. 2015) and reduces to <3 Hz with three locations (Holcombe and Chen 2013). The consensus here is that there is a global 7 Hz attentional rhythm that is alternating between the cued locations and thus the sampling frequency is divided. Similarly, the attentional frequency decreases with multiple stimulus features and increasing complexity (Merholz et al. 2022). These studies highlight the important question of how to generalize these ‘attention rhythms’ to a real-world complex visual scene.

Research attempting to branch out from commonly reported visuo-spatial search paradigms have shown attentional sampling in the theta range for visual working memory (Peters et al. 2021; Pomper and Ansorge 2021; Chota et al. 2022) and visual integration (Johannknecht et al. 2024).

**Figure 4:**
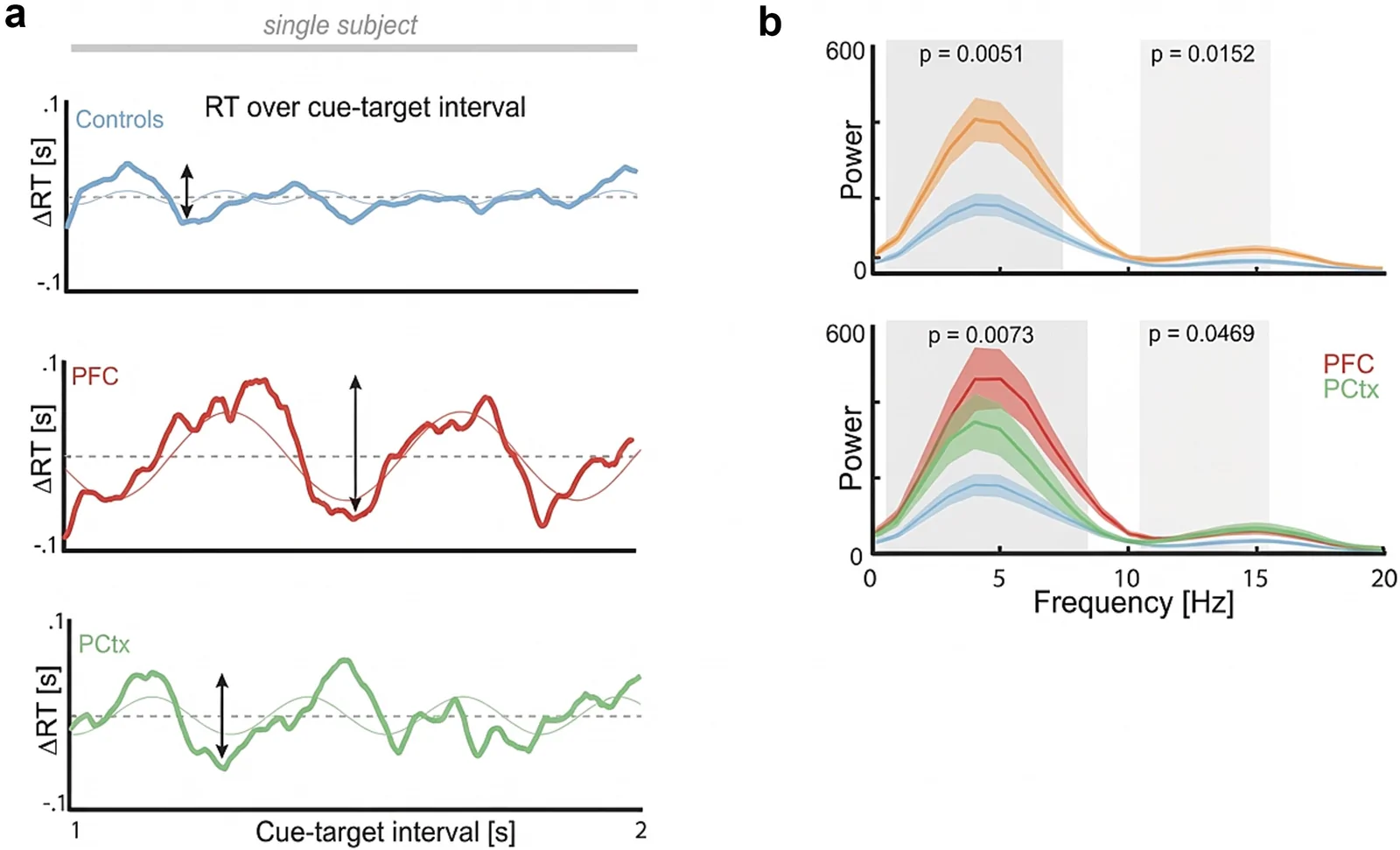
Adapted from Raposo et al. (2023). Frontoparietal lesions amplify rhythmic attentional sampling in theta and alpha/beta ranges. Participants fixated centrally while attending to a cued hemifield (70% validity) and detected a target (blue square) after a variable cue–target interval (1000–2000 ms). (a) Demeaned, time-resolved reaction times (RTs) for a representative participant from each group: Controls (blue), prefrontal cortex (PFC) lesion patients (red), and parietal cortex (PCtx) lesion patients (green). Thin lines indicate the best-fitting unconstrained sine waves. (b) Group-level 1/f-corrected power spectra of behavioural time courses. Patients showed enhanced rhythmic fluctuations at theta (2–7 Hz, d = 0.89) and alpha/beta frequencies (12–16 Hz, *d* = 0.83).

The best causal evidence to date for rhythmic attentional cycles comes from a study investigating individuals following a stroke (Raposo et al. 2023), who suffered lesions in the prefrontal (PFC) or parietal cortex (PCtx). The authors found that whilst both stroke patients and healthy controls exhibited oscillations in their reaction times as a function of variable cue-to-stimulus intervals, stroke patients showed this to a greater degree. PFC patients showed stronger theta (2–7 Hz) rhythmic sampling, and PCtx patients showed stronger alpha/beta (12–16 Hz) rhythmic sampling (Fig. 4). Patients had more variable reaction times, and this variation was explained by worse reaction times (and on-par best reaction times). Patients also exhibited high-amplitude low-frequency oscillations over lesion sites which were correlated to the oscillation in the behavioural time course. Therefore, these lesion-induced low-frequency oscillations appear to disrupt inherent theta and alpha oscillations which support attention sampling. This collection of results provides a causal link between brain oscillations in the frontoparietal network and behaviourally derived oscillations in perception. Furthermore, this account is consistent with the suggestion that frontal areas are associated with theta rhythmic sampling and posterior areas with alpha.

Fiebelkorn and Kastner (2019) have proposed an expansion to this discrete perception framework. They suggest that attentional sampling occurs at theta frequency, and that this organizes neural activity which supports sensory or motor processes into diametrically opposed phases. This provides opportune windows for attention sampling (sensory) and attention shifting (motor) at opposite theta phases. Their ‘rhythmic theory of attention’ posits that areas in the attention network that are functionally implicated in both spatial attention and saccades, may be governed by this theta-rhythmic sampling-shifting mechanism. Under this account, perception is discretized at theta frequency, however, the ‘gaps’ in perception are conceived as opportunities to reorientate attention, providing a plausible basis for adaptive behavioural flexibility.

It is worth noting that the large majority of this research is restricted to the visual modality. Applying these research paradigms to non-visual modalities is more challenging since the onset and offset times of other stimuli is harder to control. Additionally, the auditory system’s tendency to entrain to a stimulus frequency is a methodological concern. However, preliminary research examining auditory and somatosensory perception has reported similar frequency ranges to the visual research (VanRullen 2016, 2018).

An additional and complementary window into rhythmic perceptual organization comes from bistable and multistable perception paradigms, such as binocular rivalry and the Necker cube, where perception alternates spontaneously between competing interpretations despite constant sensory input. Unlike near-threshold detection paradigms, bistable perception probes the temporal stability of perceptual states rather than momentary access. Recent work has shown that individual differences in perceptual alternation rates are systematically related to properties of ongoing neural oscillations, particularly within the alpha band. Specifically, slower individual alpha peak frequency is associated with longer perceptual dominance durations and more stable perceptual cycles (Katyal et al. 2019; Torralba Cuello et al. 2022; Sponheim et al. 2023). These findings suggest a scale-consistent relationship between oscillatory temporal structure and perceptual stability, whereby slower intrinsic neural rhythms support longer windows of perceptual integration. Importantly, this relationship appears to extend across timescales, linking sub-second oscillatory dynamics to perceptual alternations unfolding over several seconds. Bistable perception thus provides converging evidence that neural oscillations do not merely gate discrete perceptual samples, but also shape the temporal persistence of perceptual states, offering a mechanistic bridge between oscillatory dynamics and the phenomenology of perceptual continuity.

VanRullen offers functional explanations for the occurrence of perceptual cycles: (i) that is energetically efficient to organize perception in this way, (ii) that it aids cross-neural and cross-frequency communication, or (iii) that there is no functional advantage and it occurs as a consequence of satisfying physiological and anatomical constraints (VanRullen 2016). The present article adds to this discussion by recognizing respiration as a lower frequency oscillation which could temporally organize this perceptual oscillation hierarchy (i, ii). In addition, this article explores the last stance here (iii), that it is a fundamental energetic principle for perception to occur in discrete moments, due to the governing role of prana.

## Prana, pranah, and perception

To complement the neurophysiological account, it is instructive to consider yogic perspectives on vital energy (prana) and its role in perception and attention. While originating in a different epistemological framework, these descriptions align conceptually with the present framework, with an emphasis on rhythmic gating of perception and attentional stability.

In yogic philosophy, prana is a foundational concept often translated as ‘energy’. Within this tradition, prana is described as a fundamental principle underlying both material organization and vital processes. According to yogic texts, the material world is understood to emerge through a process termed saiṋcara, in which subtle consciousness progressively densifies (Saiṋcara and Práńáh, Anandamurti 1959). Within this framework, material structures are described as being shaped by the interaction of opposing tendencies: centripetal (interial) forces that maintain cohesion and centrifugal (exterial) forces that promote dispersion. The dynamic balance of these tendencies is collectively referred to as prana.

When these principles are applied to living organisms, yogic texts distinguish between prana as a general energetic principle and pranah as ‘vital energy’, that is, energy specifically operative within biological life (Life, Death, and Saḿskára, Anandamurti 1959).

According to yogic psychology, pranah underlies all vital functions of the body and mind, most of which operate outside of conscious awareness. Of particular relevance to the present framework is the claim that pranah plays a central role in regulating mental stability and perceptual clarity. Acting at the interface between mind and body, pranah is described as facilitating both the reception of sensory information and the execution of motor actions:

*‘Indriyánáḿ manonáthah manonáthastu márutah.’* [The mind is the lord of the indriyas. The vital energy is the lord of the mind.] (Ekendriya - 6, Anandamurti 1988)

There are 5 sensory organs and 5 motor organs, termed the 10 ‘indriyas’. Sensory indriyas are the eyes, ears, nose, tongue and skin. Motor indriyas are the vocal chords, hands, feet, anus, and genitals (Chapter 2, Anandamurti 1962).

Within this schema, the 10 indriyas—5 sensory (eyes, ears, nose, tongue, skin) and 5 motor (speech, hands, feet, excretory, and reproductive organs)—serve as channels of interaction between the organism and its environment (Anandamurti 1962). Yogic texts propose that sensory stimulation alone is insufficient for perception to arise; rather, perception requires facilitation by pranah. Similarly, motor intentions formed in the mind require pranah for execution. In this sense, pranah is described as operating at the ‘gateway’ between sensation, cognition, and action (Práńáyáma Sádhaná, Anandamurti 1958).

A defining feature of pranah in yogic theory is its pulsative or rhythmic nature. Classical texts describe energy not as flowing continuously, but as unfolding in discrete pulses:

Energy in motion is not continuous but flows in definite little jumps; thus the stream of energy has been called systaltic or pulsatory in the scriptures. (The Chariot and the Charioteer, Anandamurti 2021)

This pulsative model is used to explain how discrimination and perception are possible: if energetic processes were entirely continuous, differentiation between objects or events would not occur. Instead, alternating phases of motion and pause are proposed to enable both the accumulation and expression of activity. Within this framework, sensory assimilation is said to occur preferentially during pause phases, whereas motor action is associated with phases of motion (Práńáyáma Sádhaná, Anandamurti 1958; Shiva—the Focal Point of Everything, Anandamurti 1982).

Applied to mental function, pranah is thus described as acting as a pacemaker for the dynamics of attention and perception. When pranah is predominantly in motion, sensory receptivity may be disrupted by ongoing activity. During pause phases, relative quiescence is said to allow incoming sensory information to be assimilated into conscious experience (Mind, Práńendriya, and Vrtti, Anandamurti 1959). Yogic texts further propose that pranah contributes not only to basic perception but also to subsequent processes of abstraction and subjectification, giving rise to qualitative experience such as softness or harshness, pleasantness, or aversion (Anandamurti 1958, 1959, 1962).

From this perspective, pranah governs the rhythm of the mind through its pulsative dynamics, with the duration and regularity of pause phases influencing perceptual clarity and cognitive stability. Importantly, yogic practice holds that humans can volitionally influence these dynamics, most notably through pranayama—the regulation of pranah.

Pranayama is described as the practice of bringing vital processes that are typically unconscious under increasing degrees of conscious control (Questions and Answers on Meditation, Anandamurti 1991). Two primary routes are described. The first is dharana, or sustained mental concentration, through which focused attention is said to stabilize pranah indirectly. The second, considered more accessible, involves the regulation of breathing. Breath occupies a unique position within yogic physiology: it is both autonomic and voluntarily controllable, and it is tightly coupled to cardiovascular and metabolic processes. As such, regulating the breath is proposed to produce reliable changes in both physiological state and mental stability.

Yogic texts repeatedly note a bidirectional relationship between breathing and mental state:

When the breath wanders the mind is unsteady, but when the breath is calmed, the mind too will be still. (Hatha Yoga Pradipika, Swami Svatmarama 1999)

When the human mind is calm, the respiration slows down. But conversely, when the mind is agitated, the breathing becomes rapid. (Ekendriya - 6, Anandamurti 1988).

…whenever people ponder something with rapt attention, the movement of their vital functions gradually becomes more and more tranquil… (Práńáyáma Sádhaná Anandamurti 1958)

Whenever you are doing something crude, your respiration becomes very active; and when you are thinking of something subtle, it becomes slow, extremely slow. (The Cult of Spirituality—the Cult of Pinnacled Order, Anandamurti 1991)

Empirical studies support the yogic observation that a calmer mind is associated with slower and more regular respiration. Long-term mindfulness meditation is associated with slower baseline respiration rates (Wielgosz et al. 2016), and this effect is also observed when accounting for meditation depth, indicating that respiration rate decreases as meditation becomes more stable or concentrated (Katyal and Goldin 2021a).

The above observations align with the yogic claim that pranayama operates by expanding the duration of pause phases in pranah, thereby increasing receptivity and stabilizing attention:

The practice of práńáyáma is the practice of control of the práńa - of the expansion of the period of pause for the maximization of the power of concentration and receptivity. (Práńáyáma Sádhaná Anandamurti 1958)

Breath regulation may thus influence perception by reducing the frequency of motion phases that interfere with sensory assimilation, while increasing the regularity and predictability of receptive periods. Yogic practices manipulate breath rate, depth, inhalation–exhalation ratios, retentions (kumbhaka), posture, and synchrony with movement (Anandamurti 1958, 1967, 1999). While advanced pranayama techniques are traditionally taught under expert guidance, regulating habitual breathing—slowing, deepening, and regularizing the breath—is presented as a safe and widely applicable method for stabilizing pranah and mental activity.

In summary, yogic models propose that perception and cognition unfold rhythmically, governed by the pulsative dynamics of pranah. Sensory assimilation is said to occur preferentially during pause phases, while motor activity corresponds to phases of motion. Through pranayama, individuals may lengthen and stabilize these pause phases, thereby enhancing perceptual clarity and attentional stability. Within the framework developed in this review, these descriptions provide a first-person, phenomenological account that complements neurophysiological models of rhythmic perception and respiratory–neural coupling, without asserting a direct physical ontology for prana itself.

## Discussion: towards the REP Framework

We propose the REP Framework, in which respiration acts as a rhythmic scaffold that structures conscious experience by modulating cortical oscillations, gating perceptual windows, and stabilizing attention. This framework integrates three interdependent levels:

(i) Neurophysiological level: Respiration entrains cortical oscillations *via* cross-frequency coupling, influencing excitability patterns across the brain.
(ii) Perceptual–phenomenological level: Oscillatory dynamics create transient pause states, which probabilistically gate sensory perception and shape perceptual rhythms.
(iii) Regulatory/attentional level: Volitional control of breathing stabilizes oscillatory and perceptual dynamics *via* neuromodulatory systems such as the LC, enhancing attentional stability and perceptual precision.

**Figure 5:**
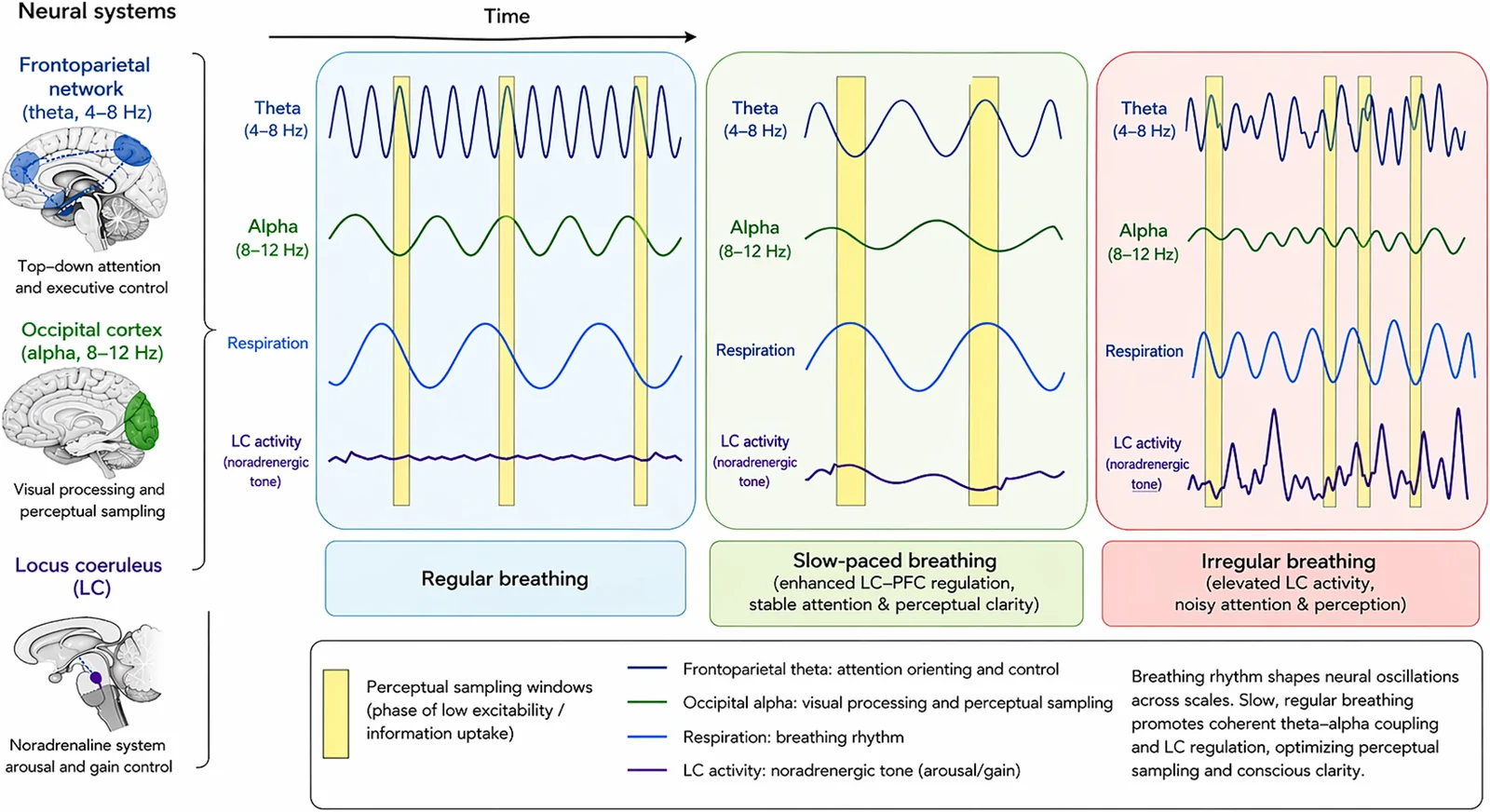
Respiration–LC–cortical coordination shapes probabilistic perceptual pause states. Schematic representation of interactions between respiratory rhythm, tonic locus coeruleus-noradrenaline activity, and cortical oscillatory dynamics frontoparietal theta and occipital alpha activity. Yellow shaded regions indicate perceptual pause states—low-excitability phases of cortical oscillations during which perceptual access is probabilistically enhanced. These pause states occur frequently in ongoing brain activity; their depiction here is intentionally simplified to illustrate relative timing, regularity, and duration rather than absolute frequency or exclusivity. Under regular breathing, tonic LC activity remains relatively stable, and cortical pause states emerge at fairly regular intervals, shown here as biased toward the late expiratory phase. During slow-paced breathing, tonic LC activity becomes more rhythmic and synchronized with respiration, resulting in longer and more temporally stable pause states that preferentially occur during extended late-exhalation periods. In contrast, under irregular breathing, tonic LC fluctuations become weakly coupled or uncoupled from respiratory oscillations, leading to pause states that are temporally fragmented, variable in duration, and less consistently aligned with respiratory phase. Overall, the figure illustrates how breathing patterns modulate the probability, timing, and stability of perceptual pause states *via* neuromodulatory and oscillatory mechanisms, rather than imposing deterministic gating of perception.

Together, these levels constitute a single, hierarchical system in which bodily rhythms, neural dynamics, and attention interact continuously to shape conscious experience. Using the REP framework, we outline how regular, slow-paced, and irregular breathing patterns are predicted to influence perception-linked brain oscillations, the probabilistic gating of perceptual-access pause states, and LC-mediated arousal and attentional regulation (Fig. 5). In Table 1 a full summary of key discussion points and hypotheses, are provided.

**Table 1:** Core hypotheses, testable mechanisms, experimental directions, and clinical and translational hypotheses, collated and summarized from the discussion

Level	Key points
**Core hypotheses**	Sensory detection occurs during transient neural ‘pause states’ (low-excitation phases of alpha/theta). Perceptual access favoured during internal quietness with minimal competing signals. Slow breathing increases duration and stability of pause states, enhancing attention and perception. Respiration entrains neural oscillations, shaping when excitatory windows open and close. Motor and sensory processes are temporally opposed: perception in pause phases, motor execution in motion phases. Disrupted rhythmic gating or irregular breathing may lead to hallucinations or attentional fragmentation.
**Testable mechanisms**	Cross-frequency coupling links respiration to cortical alpha/theta rhythms. Pause states = low-power phases, opening perceptual windows. Slow breathing stabilizes cortical excitability and attentional focus *via* neuromodulatory systems (e.g. LC). Respiration modulates phase and amplitude of perception-linked oscillations. Breathing dynamics (rate, inhale/exhale ratio, holds) influence timing and duration of pause states.
**Experimental Directions**	Test how respiration modulates alpha/theta power and phase. Assess alignment of pause states with perceptual sensitivity. Explore interactions between slow breathing and meditative concentration. Examine effects on bistable perception, attentional switching, and sustained focus. Investigate coupling between respiration, LC activity, and cortical excitability.
**Clinical/Translational Hypotheses**	Respiratory interventions may restore rhythmic gating and attentional stability. Individuals prone to hallucinations may show disrupted respiratory-oscillation coupling. Slow-paced breathing/pranayama may enhance perceptual precision and cognitive control. Breathing interventions could serve as adjuncts in mental health conditions with disrupted rhythmic gating (e.g. schizophrenia, autism, PTSD).

## Neurophysiological level: respiration as a temporal modulator of oscillations

Understanding respiration’s influence on cognition requires first considering its neurophysiological impact on cortical dynamics. Neural activity is inherently rhythmic, with ongoing oscillations shaping when neurons are most or least responsive to input. Respiratory cycles, though much slower than cortical rhythms, are well positioned to act as a global temporal modulator, systematically biasing the excitability of neural circuits. Examining this coupling between breath and brain activity provides the mechanistic basis for how respiration can subsequently influence perception, attention, and higher-order cognitive functions.

Perception and attention are rhythmically structured by neuronal oscillations, particularly in the theta-alpha range (7–11 Hz). Respiratory cycles (0.1–0.3 Hz) provide a slower bodily oscillator capable of modulating cortical excitability (Zelano et al. 2016; Perl et al. 2019; Kluger et al. 2021; Kluger and Gross 2021). In line with embodied cognition, this suggests that perception should not be modelled solely as a product of cortical rhythms, but as a coupled system spanning brain and body.

From a yogic perspective, the concept of pranah offers a close conceptual analogue to this form of brain–body coupling. Yogic texts describe pranah as a vital, pulsative force that operates in parallel with mental activity and strongly influences cognitive function. Although not defined in terms of frequency or neural substrate, pranah is said to organize the functional state of the mind, such that changes in breathing reliably alter attentional clarity, stability, and perceptual capacity.

The mechanism of cross-frequency coupling offers a candidate pathway. Phase–amplitude coupling between slow and fast oscillations is a well-established feature of cortical coordination (Fries 2005; Canolty and Knight 2010; Munia and Aviyente 2019). Within this REP framework, respiration may act as a global modulatory signal that shapes the amplitude (power) and phase organization of ongoing theta–alpha activity, thereby biasing the excitability landscape of cortical networks. Importantly, respiration is proposed to modulate when oscillatory activity is more or less excitable (power changes over time), rather than altering the intrinsic frequency of the alpha or theta rhythm. Variations in respiratory phase and pace would therefore be expected to bias the temporal structure of cortical excitability, determining when neural systems are optimally responsive to incoming information. Future research should test how respiratory dynamics modulate alpha/theta power and phase across frontoparietal and occipital networks, and whether these effects provide the neurophysiological preconditions for downstream perceptual and attentional gating (VanRullen 2016, 2018).

## Perceptual–phenomenological level: oscillatory pause states gate sensory perception

If respiration shapes the oscillatory landscape of the brain (Level 1), the next question is how this modulation translates into perceptual experience. At the perceptual–phenomenological level, we propose that oscillatory dynamics give rise to transient pause states—brief periods of reduced internal interference during which sensory input is most likely to reach awareness. Conscious perception, on this view, is not continuous, but probabilistically gated by the timing of these pause states.

### Respiratory modulation of perceptual access

Empirical evidence supports the existence of respiration-linked fluctuations in perceptual sensitivity. Kluger et al. (2021) showed that visual detection thresholds vary systematically across the respiratory cycle, with enhanced sensitivity during early-to-mid inhalation. Crucially, this behavioural facilitation coincided with transient suppression of parieto-occipital alpha power. These findings suggest that respiration does not merely modulate neural activity in general, but selectively biases when perceptual access is most likely to occur.

At first glance, this result appears paradoxical. Parieto-occipital alpha oscillations have been characterized both as an attentional sampling rhythm and as an inhibitory mechanism that suppresses sensory processing when alpha power is high immediately prior to stimulus onset. However, these functions are not mutually exclusive. Alpha rhythms may discretize sensory sampling over time while simultaneously suppressing competing or irrelevant input. Sustained alpha power can therefore support sustained task engagement and reduce mind-wandering, yet momentarily hinder the detection of brief, near-threshold stimuli by maintaining inhibitory tone in sensory cortex.

Within this REP framework, transient alpha suppression marks a pause in ongoing inhibitory control, creating a window in which sensory input can be more effectively sampled. Respiration-linked alpha suppression may therefore reflect the emergence of a perceptual pause state—a brief interval characterized by reduced internal competition and heightened receptivity.

### Pause states as perceptual gates

We propose that perceptual access is favoured during these transient pause states, which arise from the intrinsic cyclic structure of neural oscillations. Oscillatory activity necessarily alternates between periods of increased neuronal firing and relative quiescence. Although oscillations also reflect subthreshold membrane fluctuations and large-scale network dynamics, their rhythmic structure naturally lends itself to gating mechanisms.

From this perspective, perception does not occur at peaks of activity *per se*, but during moments when internally generated activity temporarily subsides. Respiration-linked alpha suppression may reflect such a shift towards quiescence. While alpha suppression is often interpreted as increased excitability, excitability here should be understood as responsiveness rather than ongoing firing. A pause state may prime neural populations to respond more effectively to incoming sensory input, enabling a robust response in the subsequent active phase.

This leads to a core hypothesis at the perceptual–phenomenological level:

- Sensory detection is probabilistically gated during transient oscillatory pause states—low-excitation phases of alpha/theta activity—whose timing is biased by respiratory phase.

### Convergence with interoceptive gating and yogic phenomenology

Support for this principle extends beyond vision. Grund et al. (2022) demonstrated that tactile detection also fluctuates across respiratory and cardiac cycles, with optimal detection occurring during cardiac diastole—a phase associated with reduced interoceptive cardiac signalling. They concluded that perception is enhanced when internal bodily signals are least disruptive. Together with respiration-linked alpha suppression, these findings suggest a broader embodied principle:

- Perceptual access is favoured during moments of internal quietness, when competing neural and interoceptive signals are minimized.

Yogic phenomenology offers a strikingly similar description at the level of first-person experience. In yogic texts, pranah is described as a pulsative process with alternating phases of motion and pause, with sensory reception said to occur specifically during the pause phase. Importantly, this account does not specify neural mechanisms, but provides a phenomenological description of rhythmic gating that closely mirrors contemporary oscillatory models. Ongoing mental or vital activity is described as interfering with sensory clarity, while perception arises when this activity momentarily subsides.

This parallel does not imply equivalence between pranah and neural oscillations, but highlights a shared structural insight: perception emerges during pauses within rhythmic activity, not during continuous motion.

### Embodied gating across respiratory and cardiac rhythms

Yogic accounts further emphasize the role of the cardiovascular system in mediating the relationship between breathing, mental state, and perceptual clarity. Pranah is traditionally associated with the region of the chest and heart, and deep concentration is described as involving a decoupling between mental activity and cardiorespiratory rhythms. Anandamurti writes:

so long as the heart functions, all the afferent and efferent nerve fibres pulsate with the same rhythm. The pulsation of all the nád́iis [*energy channels*] also vibrates according to the same system. When one becomes engrossed in deep thought or dhyána, the nerve cells remain absorbed in this; their connection with the nerve fibres becomes weak. As a result, there is less pressure on the heart, as the pulse rate is reduced to some extent. As there is lesser need, the rate of breathing also becomes slower. (Práńáyáma Sádhaná, Anandamurti 1958)

From a contemporary perspective, this suggests a reduction in interoceptive interference during deep concentration, potentially aligning respiratory, cardiac, and neural rhythms in a way that stabilizes perceptual pause states. Whether such a decoupling between neural and cardiac-related signalling occurs during focused attention remains an open empirical question.

### Bistable perception: respiration and perceptual stability

Bistable and multistable perception paradigms, such as binocular rivalry or the Necker cube, provide a complementary window into rhythmic perceptual organization by probing the temporal stability of perceptual states rather than momentary access. Building on evidence that slower alpha oscillations support longer dominance durations and more stable perceptual cycles (Katyal et al. 2019; Torralba Cuello et al. 2022; Sponheim et al. 2023), recent studies indicate that respiratory dynamics can modulate these perceptual alternations. For instance, voluntary or exercise-induced changes in respiration rate have been shown to influence binocular rivalry alternation rates, with slower breathing associated with longer perceptual dominance and enhanced stability (Iwamoto et al. 2025; Yonekura et al. 2025).

These findings suggest that respiratory rhythms may scaffold bistable perception by biasing the timing of transient pause states, effectively stabilizing competing perceptual representations. In this view, inhalation and exhalation phases could preferentially align with periods of perceptual quiescence or heightened responsiveness, linking body rhythms directly to the temporal organization of conscious experience. Such respiratory modulation provides a mechanistic extension of our REP framework, demonstrating how slow bodily rhythms can influence not only discrete sensory access but also the persistence and stability of perceptual states over longer timescales.

A promising avenue for research is the combined effect of respiration and meditative concentration (dharana) on bistable perception. Meditation practices that cultivate sustained attention are associated with slower perceptual cycling (Carter et al. 2005; Sauer et al. 2012; Katyal and Goldin 2021b; Katyal et al. 2024). Investigating how controlled breathing interacts with attentional training could reveal synergistic effects on perceptual stability, providing an empirically tractable way to probe how body–mind rhythms jointly structure conscious experience. This integrated approach may also inform interventions aimed at enhancing perceptual and attentional stability through rhythmic entrainment of neural and bodily oscillations.

### Empirical predictions and future directions from Levels 1 and 2

Viewing perception as rhythmically gated by oscillatory pause states, including the modulation of bistable perceptual cycles, leads to several concrete, testable predictions:

- Assess whether moments of successful detection coincide with global or local reductions in dominant frequency power, indicative of transient neural quiescence.
- Use intracranial or single-unit recordings to determine whether perception-facilitating oscillatory phases reflect reduced firing, altered gain, or changes in network synchrony.
- Test whether respiratory phase modulates the amplitude or phase alignment of perception-linked oscillations, integrating respiratory dynamics, neural activity, and behavioural outcomes.
- Examine whether slower or controlled respiration extends dominance durations during bistable perception, and whether these effects interact with attentional states or meditative practice.
- Investigate whether meditative concentration (dharana) synergizes with respiration to slow perceptual cycling, increase stability, and enhance alignment between neural oscillatory pause states and perceptual windows.

Together, these findings and predictions support a unified view at the perceptual–phenomenological level: oscillatory dynamics create transient pause states that gate sensory access, and respiration biases the timing and regularity of these gates. In the next section, we extend the REP framework to attention and regulatory control, asking how slow breathing may stabilize these pause states over longer timescales.

## Regulatory/attentional level: slow breathing, pause expansion, and locus coeruleus coupling

### Slow-paced breathing as temporal regulation of perceptual access

If perceptual detection is gated by transient quiescent states in neural activity, then a regulatory mechanism is required to stabilize, extend, and reliably access these windows over time. Yogic traditions locate this regulatory function in breath control (pranayama), describing slow breathing as a means of expanding the pause phase of the pranic pulse—the period during which perception and clarity are said to arise.

We propose that this pause corresponds to a neurophysiological state of reduced excitatory drive within cortical networks, particularly in higher-order perceptual and attentional systems. Slow breathing may act not by changing the intrinsic frequency of alpha/theta oscillations, but by increasing the duration of low-power, quiescent phases within these rhythms. From this, we derive a central hypothesis at the regulatory level:

- Slow-paced breathing increases the duration and temporal stability of perceptual receptivity by expanding time spent in neural pause states, thereby enhancing attentional stability and perceptual clarity.

Despite extensive evidence that slow breathing induces physiological and psychological quiescence (Zaccaro et al. 2018), cognitive neuroscience has largely framed these effects in terms of vagal tone, reduced anxiety, or general calming (Kubin et al. 2006). While important, such accounts do not explain how breath regulation might fine-tune attention or perceptual precision. We suggest an additional, mechanistic pathway:

- Respiratory rhythms entrain and modulate the power and phase dynamics of neural oscillations underlying perception and attention, shaping when excitatory windows open and close for perceptual access.

If inhalation-linked alpha suppression facilitates perceptual sensitivity (Kluger et al. 2021), then slowing the respiratory cycle should lengthen these low-power quiescent phases, effectively expanding the temporal window of perceptual gating. Breath control would thus influence cognition not by changing oscillation frequency, but by modulating the duration and timing of excitability fluctuations.

### Evidence for oscillatory flexibility under slow breathing

Hsu et al. (2020) provide empirical support for this idea. Using MEG, they found that when participants breathed at a slower pace (0.125 Hz versus 0.25 Hz), alpha-band oscillations (8–14 Hz) showed less consistent phase patterns from breath to breath—especially during mid-inhalation. However, this inconsistency was systematic rather than random: phase deviations were larger but followed a reproducible structure. This suggests that slow breathing loosens the constraints on cortical phase timing, potentially offering greater flexibility for attention to reorient or reconfigure. From this, we propose a testable hypothesis:

- Slow breathing introduces structured variability into cortical oscillatory phase dynamics, creating extended and predictable windows for attentional reorganization.

Within this REP framework, mid-inhalation during slow breathing becomes a privileged temporal window for cognitive transitions. As respiratory rhythms slow, this window lengthens and becomes more predictable, without changing intrinsic oscillatory frequency, supporting both attentional stability and adaptability.

- Measure whether slower breathing increases the temporal alignment between neural pause phases (e.g. alpha troughs) and perceptual sensitivity.
- Use tasks requiring attentional switching or sustained focus to test whether slow breathing improves performance *via* neural phase modulation.
- Explore how other breath dynamics such as inhale/exhale ratio or breath holds influence the duration and timing of these windows.

This perspective extends the breath-attention link beyond arousal regulation to a neuro-temporal mechanism, whereby respiration actively shapes the timing of perceptual access. It reframes slow breathing not just as calming, but as a means to rhythmically structure consciousness through the intentional extension of perceptual quiescence.

### Attention as neuromodulatory stabilization: the locus coeruleus

While earlier sections proposed that respiration gates perceptual sensitivity *via* transient quiescent phases in cortical dynamics, attention reflects a broader regulatory mechanism—one that shapes the temporal context within which perception unfolds. Attention must be understood as a dynamic process, sustained and modulated over time. A growing body of evidence suggests that respiration may stabilize attention by rhythmically entraining neuromodulatory systems, particularly the LC, a brainstem nucleus critical for arousal and cognitive flexibility.

A dynamical systems framework by Melnychuk et al. (2018, 2021) proposes that attention emerges from the interaction of three partially autonomous oscillators: the respiratory rhythm, LC noradrenergic activity, and cortical attention networks. These systems can drift in and out of synchrony, producing fluctuating or stable attentional states depending on the strength and phase alignment of their coupling. We propose a testable extension of this model:

- Slow-paced breathing enhances attentional stability by entraining LC oscillations to respiratory rhythm, thereby constraining cortical fluctuations *via* neuromodulatory gain control.

Respiratory–pupillary coupling—with pupil diameter serving as a proxy for LC activity—has now been robustly demonstrated (Schaefer et al. 2023, 2025; Kluger et al. 2024), supporting the plausibility of this mechanism.

### Experimental evidence for LC–respiration entrainment

To test this hypothesis, we developed a Paced Auditory Cue Entrainment task (Andrews et al. 2025), where participants synchronized breath and response (*via* left/right mouse clicks) to alternating auditory tones at a slow, controlled pace (0.1–0.15 Hz). Continuous pupil recording served as an indirect index of LC activity. Findings indicated that in the slow-breathing group, pupil diameter oscillated at the pacing frequency, suggesting LC entrainment to respiration. This group also showed significantly more stable behavioural performance (i.e. fewer response errors), while the control group—who performed the clicking task without paced breathing—did not show this pattern of pupil dynamics and exhibited response arrhythmias.

These findings support a novel mechanistic hypothesis:

- Slow breathing stabilizes attention by synchronizing LC activity to a low-frequency rhythm, thereby reducing the stochastic fluctuations in cortical excitability that otherwise lead to attentional lapses.

### Long-term regulation: attractor dynamics and training effects

Moreover, within a dynamical systems framework, slow-breathing practice may alter long-term attentional dynamics. Stable attentional states can be conceptualized as basins of attraction (Ott 2006; Dénes and Makay 2011), and repeated breath training may deepen these attractor basins, leading to more rapid and effortless access to focused awareness. This may occur *via* strengthening of frontal executive networks, vagal tone, or increased insular interoceptive sensitivity, overall improving respiratory-LC-cortical coupling (Melnychuk et al. 2018). This view opens multiple lines of empirical investigation:

- Does slow-paced breathing strengthen coupling between LC activity and frontoparietal attentional oscillations? This could be measured *via* pupil-respiration phase-locking in tandem with EEG/MEG indices of cortical phase stability.
- Does stronger LC-respiration coupling predict increased duration or frequency of perceptual pause states? This could be assessed by correlating physiological metrics (e.g. LC-coupled oscillatory gain) and extended to coupling with behavioural markers (e.g. improved detection of near-threshold stimuli).
- Slow breathing may provide a non-invasive lever for experimentally entraining LC dynamics and testing their causal role in attentional stability.

At the regulatory level, respiration influences attention not simply by calming the organism, but by entraining neuromodulatory and cortical rhythms that stabilize the temporal structure of perception. Slow breathing emerges as a means of rhythmically organizing consciousness—extending perceptual pause states, stabilizing attentional dynamics, and aligning brain–body oscillations into a coherent system. This situates breath regulation as a core mechanism of attentional control, bridging yogic theory and contemporary neurocomputational models.

## Extensions of the REP Framework

### Motor and sensory rhythms in opposition: functional partitioning within a shared cycle

While this review has focused on respiration’s role in shaping sensory perception and attentional stability, the same rhythmic framework offers a principled account of how perception and action are temporally coordinated. Rather than constituting a separate mechanism, motor–sensory alternation can be understood as an extension of rhythmic gating within the same embodied system.

In neuroscience, Fiebelkorn and Kastner (2019) proposes that sensory sampling and motor execution are not continuously available but alternate across opposite phases of a theta rhythm (≈4–7 Hz). In this model, perceptual sensitivity and motor readiness are temporally segregated, preventing interference between input sampling and output execution.

A closely aligned claim appears in yogic theory. Anandamurti describes sensory internalization as occurring during the pause phase of the pranic cycle, while motor externalization unfolds during the motion phase (Shiva—the Focal Point of Everything, 1982). Perception and action are thus treated not as concurrent processes, but as complementary phases of a single oscillatory system, each supported by distinct physiological states.

When integrated with the present REP framework, these views converge on a testable extension:

- Motor and sensory processes are temporally gated by respiration, in opposition, such that perceptual receptivity is optimized during the quiescent pause phase, while motor execution is facilitated during the active motion phase.

Empirical findings are consistent with this antagonistic pattern. Johannknecht and Kayser (2022) examined six sensory–cognitive domains and found that stimulus presentation tended to align with inhalation, whereas motor responses were more likely during exhalation. This task–respiration synchrony suggests that respiratory phase may be adaptively aligned with behavioural priorities.

Similarly, Perl et al. (2019) reported that participants in a visuospatial attention task preferentially self-initiated trials at inhalation onset, consistent with optimizing perceptual sensitivity. In contrast, Park et al. (2020) found that self-initiated motor actions unlinked to sensory input were more likely during exhalation. Together, these findings suggest a flexible respiratory coupling that prioritizes perception or action depending on task demands.

Yogic theory further extends this partitioning beyond immediate perception and action. Pranah is also proposed to support post-perceptual processes such as abstraction, rule formation, and mental synthesis (Anandamurti 1958, 1959, 1962). These processes may unfold across multiple motion–pause cycles, plausibly reflecting recurrent neural activity as information propagates through distributed cortical networks.

This raises a broader empirical question central to the framework:

- How does the brain dynamically align respiratory phase with the dominant demands of a task (sensory, motor, or cognitive)?

Different experimental designs place different weights on detection, discrimination, decision-making, and response execution. Respiratory phase alignment may therefore vary accordingly, potentially explaining inconsistencies across studies. This motivates a methodological recommendation:

Future perception studies should minimize immediate motor responses (e.g. delayed or block-wise reporting) to dissociate respiratory modulation of perception from motor-related respiratory entrainment. Such designs would help isolate the sensory gating role of respiration more cleanly, strengthening tests of the core REP framework.

### Mental health and rhythm disruption: perceptual instability as a failure of rhythmic gating

A further extension of the REP framework concerns mental health conditions characterized by perceptual and attentional anomalies. If perception depends on properly timed pause states within a coordinated brain–body rhythm, then disruptions to this rhythm should produce characteristic perceptual distortions.

One potential locus of dysfunction is impaired intracortical rhythmic sampling. For example, frontal and parietal stroke lesions have been shown to induce aberrant low-frequency oscillations that degrade perceptual awareness (Raposo et al. 2023). From a rhythmic perspective, this may reflect a breakdown in the timing or reliability of perceptual gating windows, leading either to excessive sensory noise or to failures of access.

Yogic theory offers a phenomenological parallel. Anandamurti describes perception as emerging from oscillatory ‘ectoplasmic waves’ characterized by alternating motion and pause. He suggests that perceptual distortions arise when this rhythmic integration becomes imbalanced:

The flow of ectoplasmic waves moves continuously on, and those waves are also systaltic, with crests and troughs, speed and pause... When there is a positive tendency in the mind, it integrates everything which it receives and claims that it has seen an object... Now suppose that there is not a positive tendency but a negative tendency... in that case, though the mind projects an elephant within, we will not see it... Because of this psychic defect or disorder, we have hallucinations, sometimes positive and sometimes negative. (Reality and Intellectuality, Anandamurti 1987)

Here, positive hallucinations (perceiving absent stimuli) and negative hallucinations (failing to perceive present stimuli) are attributed to mismatches between internal dynamics and external input. Translated into contemporary terms, this aligns closely with predictive coding accounts in which hallucinations arise from misweighted top-down predictions relative to sensory evidence (Sterzer et al. 2018).

Within the present framework, this suggests:

- Hallucinations and perceptual distortions may arise from disrupted phase-locked rhythmic gating, leading to misalignment between sensory input and cognitive expectation.

Respiration may play a contributory role in such dysregulation. Brændholt et al. (2023) report atypical respiratory patterns in conditions including schizophrenia, autism, and PTSD, and propose that irregular breathing may perturb respiratory–neural coupling. From a rhythmic gating perspective, irregular respiration could destabilize the temporal structure of theta/alpha oscillations, impairing the precision of perceptual sampling.

This leads to a mechanistic pathway:

- Irregular breathing may produce erratic perceptual gating, increasing susceptibility to hallucinations, derealization, or attentional fragmentation by disrupting oscillatory timing.

Crucially, this framing implies a novel therapeutic implication:

- Could respiratory interventions restore rhythmic regularity in the motion–pause cycle, thereby stabilizing perceptual gating and attentional control?

Slow-paced breathing and other forms of pranic regulation may help re-establish the temporal scaffolding of perception by modulating cortical oscillations, neuromodulatory systems (e.g. LC), and interoceptive signals. These practices could therefore be explored as low-cost, accessible adjuncts to existing treatments.

Key empirical questions emerging from this extension include:

- Do individuals prone to hallucinations exhibit atypical theta/alpha phase-locking or phase–amplitude coupling during sensory tasks?
- Are such neural irregularities correlated with disrupted respiratory rhythms?
- Can slow-paced breathing restore perceptual gating and reduce prediction error?
- Are improvements in perceptual stability associated with increased respiration–oscillation phase coupling?

### Relation to the REP Framework

Importantly, motor–sensory partitioning and mental health implications do not introduce new principles. They follow directly from the core REP framework established in Levels 1–3, once perception and attention are understood as rhythmically gated processes within an embodied dynamical system. These extensions illustrate how a single organizing principle—respiratory modulation of oscillatory pause states—can account for phenomena ranging from action selection to psychopathology. For a full summary of discussion points and hypotheses (see Table 1).

## Aligning the REP Framework with contemporary yogic epistemologies

The REP Framework aligns with a growing body of scholarship that seeks to integrate ancient contemplative insights with contemporary cognitive science. By positioning respiration as a rhythmic scaffold for consciousness, the REP framework provides a physiological mechanism for several established theoretical models.

The REP framework provides a neurobiological substrate for the Yogic Theory of Consciousness (YTC) formulated by Tripathi and Bharadwaj (2021). While YTC distinguishes between the emergent ‘mind’ (intellect, ego, and sensory processing) and the fundamental ‘seer’ (witnessing awareness), it posits that the mind is typically obscured by various modulations (vrittis) such as sleep, memory, and mind-wandering. The REP framework offers a possible operationalization of this view by proposing that respiratory-driven oscillations may act as a global temporal modulator that constrains fluctuations in cortical excitability. In this way, rhythmic breathing allows the emergent mind to align with the stable awareness of the ‘seer’.

The framework’s Perceptual–Phenomenological Level resonates with the updated Samkhya model proposed by Katyal (2022), which details four structures of consciousness: citta (mental object), ahamtattva (doer I), mahattattva (existential I), and purusha (witnessing I). Katyal describes meditation as a ‘stepwise experiential reduction’ where the practitioner eliminates these structures one by one to reach the non-dual stance. The REP framework’s concept of ‘pause states’—transient windows of reduced internal interference—mirrors this reduction. Specifically, the REP framework suggests that respiratory-linked modulation of alpha/theta power and phase-dependent excitability, alongside the lengthening of low-interference (‘pause’) phases, could provide the ‘internal quietness’ required to momentarily suspend doership (ahamtattva) and expand the window of pure existential awareness (mahattattva).

The Regulatory/Attentional Level of the REP framework provides a mechanistic account of the Cognitive Training Hypothesis reviewed by Sedlmeier and Srinivas (2016). They argue that yoga practice leads to ‘sattvification’—a shift towards mental purity, stability, and harmony. The REP framework proposes that slow-paced breathing is the physiological ‘lever’ that facilitates this state by expanding the duration of neural pause states and increasing attentional stability. This aligns with the Samkhya-Yoga personality theory, where a dominant Sattva Guna is associated with high stress tolerance and a calm, disciplined mind.

Finally, the REP framework bridges the gap between theories of discrete perception and the phenomenological experience of continuity. Katyal (2022) distinguishes between dharana (concentration on discrete, static mental images) and dhyana (meditation as a ‘dynamic force or unbroken flow). While cortical rhythms (theta/alpha) support the discrete sampling of attention, the REP framework suggests that the slower respiratory rhythm acts as a continuous scaffold. By entraining faster cortical oscillations, respiration unifies these discrete samples into the structured temporalities of experience described in both contemporary neuroscience and ancient yogic phenomenology.

## Conclusion: the REP Framework

Across neurophysiological, perceptual–phenomenological, and regulatory–attentional levels, respiration emerges as a core temporal organizer of brain and behaviour. We term this the REP Framework, which conceptualizes breathing as a multi-level scaffold linking body and brain while highlighting perception’s rhythmic structure. At the neurophysiological level, slow respiratory cycles act as a global modulatory rhythm, shaping the phase and excitability of faster cortical oscillations. These dynamics provide the mechanistic substrate through which perception and attention are rhythmically gated, linking body and brain into a coupled system. At the perceptual–phenomenological level, oscillatory pause states—periods of transient cortical quiescence—create temporal windows for sensory access. Respiration biases the timing and duration of these windows, coordinating moments of perceptual clarity and attentional receptivity. Empirical evidence from vision, touch, and interoception supports this rhythmic gating, while yogic descriptions of the pranic pulse offer a convergent first-person account, highlighting the functional significance of pauses within ongoing activity. At the regulatory–attentional level, slow-paced breathing stabilizes these pause states, extending perceptual windows and enhancing attentional continuity. Respiratory entrainment of the LC and associated neuromodulatory systems further constrains cortical fluctuations, reducing stochasticity and supporting cognitive stability. Repeated breath training may deepen attractor states of focused awareness, suggesting that respiration can act as a non-invasive tool for shaping long-term attentional dynamics.

Extensions of the REP framework illustrate its broader explanatory power. Sensory and motor processes appear temporally partitioned, alternating across respiratory phases to prioritize perception or action, while disruptions in respiratory–neural coupling may contribute to perceptual instability and psychopathology. Together, these observations suggest a single organizing principle: respiration provides a rhythmic scaffold that coordinates neural excitability, perceptual access, attentional stability, and behaviour.

Looking forward, the REP framework offers multiple avenues for empirical exploration: mapping respiration–oscillation coupling across cognitive domains, testing the causal impact of slow breathing on attentional dynamics, and evaluating its therapeutic potential in conditions marked by disrupted rhythmic gating. By linking body, brain, and mind through temporally structured rhythms, this perspective unifies ancient yogic insight with contemporary neuroscience, highlighting respiration as a central mechanism of cognitive organization and well-being.

## Data Availability

No new data is available from this manuscript.
