# Correction to: The Rhythmic Embodied Perception Framework of breath, brain, and perception

**DOI:** 10.1093/nc/niag076

**Published:** 2026-10-23

This is a correction to: Ralph Andrews, Paul M Dockree, The Rhythmic Embodied Perception Framework of breath, brain, and perception, *Neuroscience of Consciousness*, Volume 2026, Issue 1, 2026, niag030, https://doi.org/10.1093/nc/niag030

In the originally published version of this manuscript, Figures 1 and 2 were erroneously switched.

This error has been corrected.

